# Breastfeeding among Hispanic and Black Women: Barriers and Support

**Published:** 2023-08-31

**Authors:** Sarah G Buxbaum, Olumide Arigbede, Arlesia Mathis, Fran Close, Selina F Darling-Reed

**Affiliations:** College of Pharmacy and Pharmaceutical Sciences, Institute of Public Health, Florida A&M University, Tallahassee, FL, USA

## Abstract

We describe barriers and supports for the practice of breastfeeding, with particular focus on Black and Hispanic women in the United States. We note that breastfeeding patterns reported by WIC agencies is highly variable across the country and within states. The global campaign to support breastfeeding, Baby Friendly Hospital Initiative, and its implementation in the US is described, as well as Healthy People goals and the mixture of policies across the US that provide incomplete support for breastfeeding mothers.

## Introduction

Breastfeeding has health advantages for both the breastfed infant and the breastfeeding mother. The nursing pair is referred to as the breastfeeding dyad, and the advantages to the child in the dyad are wide-ranging, from clinical endpoints such as morbidity and mortality, to more nuanced effects such as cognitive and emotional intelligence, e.g., resilience. Human breast milk is a source of complete nutrition for a human infant that costs little to provide [1]. Documented advantages to breastfeeding mothers include reduced breast and ovarian cancer morbidity, reduced incidence of anemia and osteoporosis, and there are likely nuanced impacts on their well-being due to hormonal changes and the relationship building that breastfeeding supports.

Breastfeeding benefits both newborns’ and young children’s health and survival. Recent studies indicate that Exclusive Breastfeeding (EBF) in the first 6 months of life lowers morbidity and mortality associated with infectious disorders such as diarrhea and respiratory infections, resulting in fewer hospitalizations for babies and young children and saving the lives of approximately 820,000 children under the age of 5 years annually [2]. Other studies show benefits to both mother and child in terms of nutrition, physiology, and development [2,3]. Substantial differences in cognitive results, mother-child attachment, and resilience between breastfed and bottle-fed infants have been documented [4,5].

## Health Disparities in Breastfeeding in High-income Nations

Although breastfeeding is less expensive and of greater nutritional value than purchasing supplements, in high-income nations, newborns from impoverished households are less likely to be breastfed than infants from more advantaged families. Consistent with trends in the United States (US), evidence from other developed nations such as Australia and Canada suggests that the economic gap between the affluent and poor in industrialized countries is increasing, thereby contributing to health disparities suffered by low-income families [6]. Similarly, there is a difference in breastfeeding behavior according to economic status. In the US, women selected for participation in the Women, Infant and Child Program (WIC) have lower rates of initiation than those not selected for WIC; however, these differences can be ameliorated with educational programs about breastfeeding [7]. Disparities in breastfeeding behavior matter because there are well-documented health advantages of breastfeeding to the mother which include but are not limited to reduced risk of cancer and type 2 diabetes, and increased birth spacing which gives the mother time to recuperate [8]. Breastfeeding also provides advantages to the infant such as positive effects on the infant’s emotional intelligence and attachment, orofacial structure, and immune system health, as well as reducing the likelihood of many childhood illnesses [1].

## Breastfeeding Goals in the United States

In the US, the Healthy People 2010 initiative set the following breastfeeding goals: 75% of all mothers of newborns initiating breastfeeding, with 50% continuing for at least 6 months postpartum and 25% continuing for a year, as well as 40% exclusively breastfeeding for 3 months and 17% exclusively breastfeeding for 6 months. The only goal that was accomplished was to have 75% of new mothers initiate breastfeeding [9]. Nevertheless, the HP2020 set a goal that 81.9% of infants are ever breastfed, with 60.6% continuing at least 6 months, 34.1% of infants breastfed at 1 year, 46.2% of infants breastfed exclusively through 3 months, and 25.5% exclusively breastfed up to 6 months old. The United States has set goals for Healthy People 2030 to increase the proportion of infants that are exclusively breastfed at 6 months to 42.4% and continued breastfeeding through 12 months to 54.1%. Although breastfeeding initiation rates are high in the U.S., most women do not exclusively breastfeed for 6 months nor continue breastfeeding through the first 12 months [10].

According to the Healthy People 2020 (HP2020) report, the gap between breastfeeding behavior and Centers for Disease Control and Prevention targets has widened. Asian women are the only racial/ethnic group currently reaching the Healthy People 2020 target of breastfeeding initiation, while Hispanic women are close behind, with subgroups of Hispanic women attaining this goal. In comparison to all other racial/ethnic groups in the United States, Black women had the lowest rates of both breastfeeding initiation and continuation [11].

## The WIC Program in the United States

In the US, mothers who breastfeed at a lower rate are more likely to be young, low-income, African American/Black, with “divorced, single or widowed” marital status, and less educated, overweight or obese before pregnancy, and more likely to report their pregnancy was unintended [12]. Similarly, participants in the Supplemental Nutrition Program for Women, Infants, and Children (WIC) are less likely to breastfeed. Known hurdles to breastfeeding may be divided into two categories: microenvironmental variables, such as community environment, family environment, birthing environment, and job/work environment, and macroenvironmental ones, such as political environments at the county, state, and federal levels. Many women continue to face significant obstacles in their job and family environments [13]. This is reflected in the HP2020 target of 38% of workplaces having lactation support programs, with only 28% of workplaces meeting that goal in 2014 [14]. Due to the confluence of these factors, even after deciding to breastfeed, mothers frequently fall short of their nursing goals [15].

The WIC program is a U.S. federal government-sponsored nutrition program for women, infants, and children, which may mitigate the effects of the factors mentioned above. Healthy meals, nutrition instruction and counseling, breastfeeding support, and referrals to health care and community resources are all provided free of charge through WIC [16]. However, programming varies across WIC centers, so some counties even within the same state may have more support for breastfeeding than others. Local cultural differences also clearly play a role. Nationally, the breastfed rate in WIC has increased slowly but steadily over a decade from 28.1% in 2011 to 34.3% in 2021 [17].

In the United States, national estimates reveal significant disparities in breastfeeding indices between non-Hispanic Black and non-Hispanic White babies [18]. Location matters as well. Based on WIC data, Arkansas, Mississippi, Alabama, Ohio, West Virginia and Oklahoma have total breastfeeding rates between 11 to 17%. On the high end, within the continental US, Texas has a rate of 57% for total breastfeeding, although its rate for fully, i.e., exclusive, breastfeeding is 7% [17]. This difference in the total and fully breastfeeding rates in Texas is consistent with reports that Hispanic mothers are more likely to use formula supplementation compared to other mothers and may not receive education to fully breastfeed in hospitals [19,20]. On the other hand, Hispanic mothers have a high rate of breastfeeding compared to most other groups. Texas’s WIC breastfeeding rates are highly variable, ranging from 21.9% to 84.5%. The relatively high rates are likely due to demographics: a high percentage of the Hispanics and Latinos in the US live in Texas [21]. Other locations within the United States with high rates of total breastfeeding are the US Virgin Islands (71%), American Samoa (69%), the Commonwealth of the Northern Mariana Islands (53%), the District of Columbia (51%), and Hawaii (50%), Vermont (49%), and New York State (48%). Florida is midrange at 37%, ranging from 21.2% to 48.3% among the agencies [17]. Other highly populated states, New York, Texas and California and Illinois have wider ranges (20.2 to 87.1%, 21.9 to 84.5%, 20.7 to 65.9% and 10.1 to 42.7%, respectively).

Although breastfeeding rates of both Black and White newborns have increased over the previous decade, racial inequities still exist [22]. U.S. legislation such as the Special Supplement Nutrition Program for Women Infants and Children (WIC) program (1972), the Family Medical Leave Act (1993), Personal Responsibility Welfare and Work Opportunity Act (1996), the Affordable Care Act (2010), Healthy People (2010) and the Surgeon General’s Call to Action to Support Breastfeeding [23] were created to improve work and community environments to support breastfeeding. The WIC program is the longest-running and most widely known program for its maternal and child health benefits. The WIC program was founded in 1972 and made permanent in 1974 [24] to provide supplemental food, nutrition education, and healthcare referrals to pregnant, breastfeeding, and postpartum women, as well as infants and children up to the age of five [25]. Although studies have been conducted on the influence of mothers’ WIC participation on newborn health outcomes, little is known about the pathways that potentially contribute to enhanced infant health [26]. More specifically, healthy maternal lifestyle behaviors such as smoking cessation, timely commencement of prenatal care, healthy weight gain, and breastfeeding practice may be beneficial to the health of the infants and can be affected by WIC participation [27].

Breastfeeding is a complicated activity [28] influenced by a variety of socioecological variables such as education, regulations (local, state, and federal), politics, and management support [29]. Given that WIC covers about half of all births in the United States, the program significantly impacts baby-feeding decisions, especially among low-income mothers [30]. In fact, WIC comes the closest to having a nationwide coordinated breastfeeding program to encourage mothers to exclusively breastfeed their babies during the first 6 months after birth and to ensure that they continue breastfeeding with infant formulas and/or foods until at least 24 months [31].

## Health Disparities and Policies Supporting Breastfeeding

Black mothers face a complex collection of circumstances that impact their reproductive attitudes and actions [32], such as breastfeeding [3], when it comes to making decisions about infant feeding and childcare. Black women are disproportionately more likely than White women to suffer from poor neonatal health outcomes, chronic disease, stress, depression, and posttraumatic stress disorder, all of which are established factors associated with decreased breastfeeding rates [33]. Low-income status, systematic discrimination, living in racially segregated areas, and other forms of racial disempowerment among Black people have been identified as underlying causes of racial health inequalities, contributing to negative health outcomes. Blacks experience more discrimination, thus incurring increased stress. Considering income, type of job or workplace, and other related factors, Black women are more likely than white women to fall close to the poverty line, to receive WIC benefits, to be unmarried, to have less education, and to become mothers at a younger age — each of which are related factors that contribute to lower breastfeeding initiation [34]. It’s not uncommon for hospital staff to have preconceptions that Black women do not intend to breastfeed, and this racial bias leads to lower breastfeeding initiation; however, education of staffcan ameliorate this bias [19,35]. Black women with low income also frequently lack the necessary interpersonal support from family and friends and work-related flexibility, all of which are linked to healthy breastfeeding habits. Relatedly, on average, Black mothers return to work two months after giving birth, which is sooner than women of other racial and ethnic groupings [34,36]. Furthermore, in the US, Black mothers are disproportionately more likely to report that their workplaces are hostile to breastfeeding [37].

Shorter maternity periods and return to less supportive work settings are just two of the critical obstacles encountered when returning to work, particularly for Black women who tend to work in lower-paying occupations [38]. Breastfeeding assistance at work is still uneven across the board for all women. Currently, only 29 states and the District of Columbia have laws specifically related to allowing breastfeeding in the workplace, and 31 states plus the District of Columbia and Puerto Rico exempt breastfeeding from public indecency laws. Each state now has laws that allow women to breastfeed but without the exemption from public indecency laws, the US as a whole lacks law that completely protects the right to breastfeed in public and at work.

There is very little in the literature especially in the US about the efficacy of the Baby-Friendly Hospital Initiative (BFHI) [39] as implemented in the United States [40]. BFHI is a global campaign by the World Health Organization and the United Nations Children’s Fund, which promotes best practice to support breastfeeding in maternity services. Worldwide, only 10% of infants were being born in BFHI designated hospitals [6] in 2017. Few hospitals carry the BFHI designation in the US; Munn et al. report in a review article that 8% of hospitals are BFHI [41]. In addition, they note that there is a dearth of information on breastfeeding duration or monitoring of prenatal and postnatal counseling. There is particularly little information about rural areas or the southeast. In Florida, for example, only 10 hospitals carried the designation in 2015 [42]; a national resource lists 26 facilities in Florida that currently have or have had the designation out of 128 maternity hospitals. There is little information in the literature about breastfeeding initiation or duration of breastfeeding in Florida. Using data from the Florida Department of Health, we were able to capture information about breastfeeding initiation [7], however, breastfeeding duration was not in our dataset.

In 2009, only two states, California and New York, had established a Model Hospital Breastfeeding Policy [1], setting a new evidence-based standard for all hospitals that provide maternity care services in the United States, based on the most current recommendations of expert breastfeeding groups. However, an assessment of these practices in New York state four years later in 2013 found that, on average, only half of the standards were being implemented [1]. Worldwide, hospitals themselves may not be implementing policies that support breastfeeding for their own employees via workplace breastfeeding accommodations. Studies have described deficiencies in the intentions to breastfeed among future healthcare workers, people who would presumably have the most information at hand compared to the rest of the population [43,44]. The attitudes of healthcare workers are likely to be conveyed to the patients. Thus education and standards are not sufficient to improve breastfeeding practices that improve early infant health outcomes. Supportive policy and knowledge must be backed up with the implementation of evidence-based breastfeeding accomodations. It is important to note that hospitals are increasingly providing written breastfeeding policy to their employees, a trend that is measured by the CDC’s Maternity Practices in Infant Nutrition and Care (mPINC) survey, administered between 2007 and 2018 [45]. Political support must be developed for expansion of the BFHI best practices to more hospitals, and ongoing quality assurance is essential, with systematic assessment of BFHI designated hospitals [44]. Baby Friendly Hospitals should provide breastfeeding support including long-term healthcare relationships at least throughout the perinatal period in a culturally sensitive manner [35].
